# Dynamic assessment for low back-support exoskeletons during manual handling tasks

**DOI:** 10.3389/fbioe.2023.1289686

**Published:** 2023-11-10

**Authors:** Xiaohan Xiang, Masahiro Tanaka, Satoru Umeno, Yutaka Kikuchi, Yoshihiko Kobayashi

**Affiliations:** Institute of Agricultural Machinery, National Agriculture and Food Research Organization (NARO), Saitama, Japan

**Keywords:** ergonomics assessment, lumbar load, manual handling task, dynamic simulation, low back pain

## Abstract

Exoskeletons can protect users’ lumbar spine and reduce the risk of low back injury during manual lifting tasks. Although many exoskeletons have been developed, their adoptability is limited by their task- and movement-specific effects on reducing burden. Many studies have evaluated the safety and effectiveness of an exoskeleton using the peak/mean values of biomechanical variables, whereas the performance of the exoskeleton at other time points of the movement has not been investigated in detail. A functional analysis, which presents discrete time-series data as continuous functions, makes it possible to highlight the features of the movement waveform and determine the difference in each variable at each time point. This study investigated an assessment method for exoskeletons based on functional ANOVA, which made it possible to quantify the differences in the biomechanical variables throughout the movement when using an exoskeleton. Additionally, we developed a method based on the interpolation technique to estimate the assistive torque of an exoskeleton. Ten men lifted a 10-kg box under symmetric and asymmetric conditions five times each. Lumbar load was significantly reduced during all phases (flexion, lifting, and laying) under both conditions. Additionally, reductions in kinematic variables were observed, indicating the exoskeleton’s impact on motion restrictions. Moreover, the overlap F-ratio curves of the lumbar load and kinematic variables imply that exoskeletons reduce the lumbar load by restricting the kinematic variables. The results suggested that at smaller trunk angles (<25°), an exoskeleton neither significantly reduces the lumbar load nor restricts trunk movement. Our findings will help increasing exoskeleton safety and designing effective products for reducing lumbar injury risks.

## 1 Introduction

Back-support exoskeletons help farmers, nurses, and industrial workers reduce lumbar burden and improve working efficiency (Kobayashi et al., 2009; Hasegawa and Muramatsu, 2013; Upasani et al., 2019). As one of the specific human-robot collaboration solutions for manual handling tasks, back-support exoskeleton should satisfy the safety requirement for both robot and humans (De Looze et al., 2016; Ajodani et al., 2018). Thus, developing safe and effective exoskeletons will enable a broad range of applications that could benefit users.

ISO 13482 states that the purpose of exoskeletons is to reduce physical workload (ISO, 2014). The safe limit for human lumbar workload averages 3.4 kN (Water et al., 1993). However, accurately evaluating exoskeleton safety is difficult owing to the complexity of human–robot interactions and the unpredictability in user movements. Moreover, exoskeletons can constrain human movement, leading to discomfort (Baltrusch et al., 2018). Performance variations at actual rehabilitation, industrial, and agriculture work sites highlight the need for a standardized and dynamic assessment method for exoskeletons (De looze et al., 2016; Omoniyi et al., 2020; Zheng et al., 2022).

Exoskeleton assessment requires obtaining lumbar load and human movement data. Human movement can be measured by optical or inertial measurement unit (IMU) motion capture systems. Several methods have been used to measure exoskeletons’ lumbar load, including directly recording the assistive force by inserting additional load cells into the exoskeleton (Abdoli-Eramaki et al., 2007; Abdoli-Eramaki et al., 2008); assuming a relationship between the magnitude of electromyographic signals and assistive forces, and then estimating the assistive forces by recording trunk muscles’ activities (Marras et al., 2000; Lamers et al., 2018; Weston et al., 2018; Koopman et al., 2019b); and establishing an exoskeleton by testing its characteristic performance beforehand (Koopman et al., 2019a). Nabeshima et al. (2018) developed a non-human testing framework to obtain lumbar torque. These methods, which estimate assistive forces, can be used with an inverse dynamic human model to calculate the actual lumbar load when using exoskeletons.

Further, several statistical methods have been employed to examine lumbar load and other key variables. These methods can be flexibly employed to scrutinize data across various temporal frames—individually, collectively, or even utilizing time-weighted averages—based on the specific demands of their research objectives. For instance, the effect of using exoskeleton on a biomechanical variable can be easily examined using the *t*-test (Abdoli-Eramaki et al., 2007; Whitfield et al., 2014; Lamer et al., 2018). Analysis of variance (ANOVA) would be suitable for evaluating the mixed effect between the testing conditions and exoskeleton modes on users (Marras et al., 2000; Abdoli-Eramaki et al., 2008; Ulrey and Fathallah, 2013; Weston et al., 2018; Koopman et al., 2019a; Koopman et al., 2019b; Poliero et al., 2020). Principle component analysis was used to select the important features and identify the differences of using exoskeletons (Sadler et al., 2011).

Except peak burden, when it is effective to use exoskeletons, and when motion is restricted are also concerned and can contribute to the risk of lumbar injury (Upasani et al., 2019; Omoniyi et al., 2020). Thus it is necessary to consider the effect of the exoskeletons not only on the timing when peak lumbar burden occurs but on the lumbar burden across the whole task. In addition it is also found the phase shift.

To the author’s knowledge, the effect of exoskeleton at the time other than the peak value in manual handling tasks has not been well-investigated. Therefore, we employed a time-series analysis method Functional data analysis (FDA), specifically functional ANOVA (FANOVA), which is designed to handle functional data such as biomechanical data, accounting for their continuous nature and temporal dependencies (Ramsey and Silverman, 2005). The FANOVA could be separated into a few steps. First, time-series biomechanical data such as the lumbar load and flexion angle were collected (Section 2.3 and Section 2.4). Second, functions were used to present the waveforms of the biomechanical variables, with B-spline the most commonly used (Ramsey and Siverman, 2005) (Section 2.5.1). Third, the obtained functions were aligned at identical timing points to obtain a representative comparison using a data registration (or data alignment) technique (Godwin et al., 2010) (Section 2.5.1). Finally, the FANOVA model was used to calculate the F-ratio between using and not using the exoskeleton (Section 2.5.2). In agriculture, FANOVA has been used to accurately estimate continuous growth trends (Xu et al., 2018) and demonstrate significant differences in various biomechanical contexts, such as lip kinematics and fatigue-induced kinematics changes (Ramsay et al., 1996; Godwin et al., 2010).

Compared to the traditional *t*-test, ANOVA, and PCA methods, FDA is better at dealing with the time-series dataset. The traditional methods usually identify vital features related to the performance of motions and injury mechanisms from the waveforms of the biomechanical data by referring to some individual points and reducing the dimensionality of the waveforms (Moudy et al., 2018). These methods are limited in that the important features are identified before the data have been analyzed. In contrast, FDA can be applied to multidimensional signals and eliminates the need for the prior identification of the relevant features (Donoghue et al., 2008). In addition, the traditional methods have difficulty finding the differences between groups, and individual differences may produce conditions that will cause timing/phase variability in the waveform (Moudy et al., 2018). For example, in different trials, the subject may not be able to reach the maximal flexions at the same time, while the FDA can help us to minimize the time difference (data alignment) among the maximal flexions, and to maintain the shape and amplitude of each curve. Using this technique, FDA can highlight the features of waveforms to reduce the timing/phase variability so that we can analyze the effect of exoskeletons on the magnitude at all timings, and it is not necessary to identify the peak value or mean value (Moudy et al., 2018). Thus, using FDA to assess the performance of an exoskeleton makes it possible to determine when the exoskeleton can significantly reduce the lumbar load or restrict human motion during a task.

Industrial exoskeleton usage requires safety considerations in high-risk scenarios, such as dynamic symmetrical and asymmetrical lifting tasks (De Looze et al., 2016; Huysamen et al., 2018). FANVOA can be a suitable method for revealing exoskeletons’ effect on key variables in each phase of these tasks, thus promoting their standardization.

Although important discrete peak and mean values of the lumbar load and kinematics factors have been studied in exoskeleton evaluations, no method is available to evaluate the effectiveness of various postures during lifting-flexion movement of the exoskeleton. Thus, this study was novel because it not only evaluated the performance of the exoskeleton when variables reached their peak values but also at all other times. This made it possible to identify the variability when using the exoskeleton throughout the entire lifting-flexion motion and assess its applicability to the entire manual handling movement.

In this study, we mainly focus on developing a FANOVA-based method to evaluate the effects of an exoskeleton on the biomechanical variables at every time point during the manual handling tasks. This analysis allows us to examine when exoskeletons can significantly reduce lumbar load and restrict motions during the task. In addition, we also proposed an exoskeleton–human model to estimate the dynamic lumbar load with the exoskeleton’s assistance. It was hypothesized that the FANOVA would demonstrate the exoskeleton, and significantly affect not only the peak biomechanical variables but also that at the other period.

## 2 Materials and methods

**FIGURE 1:**
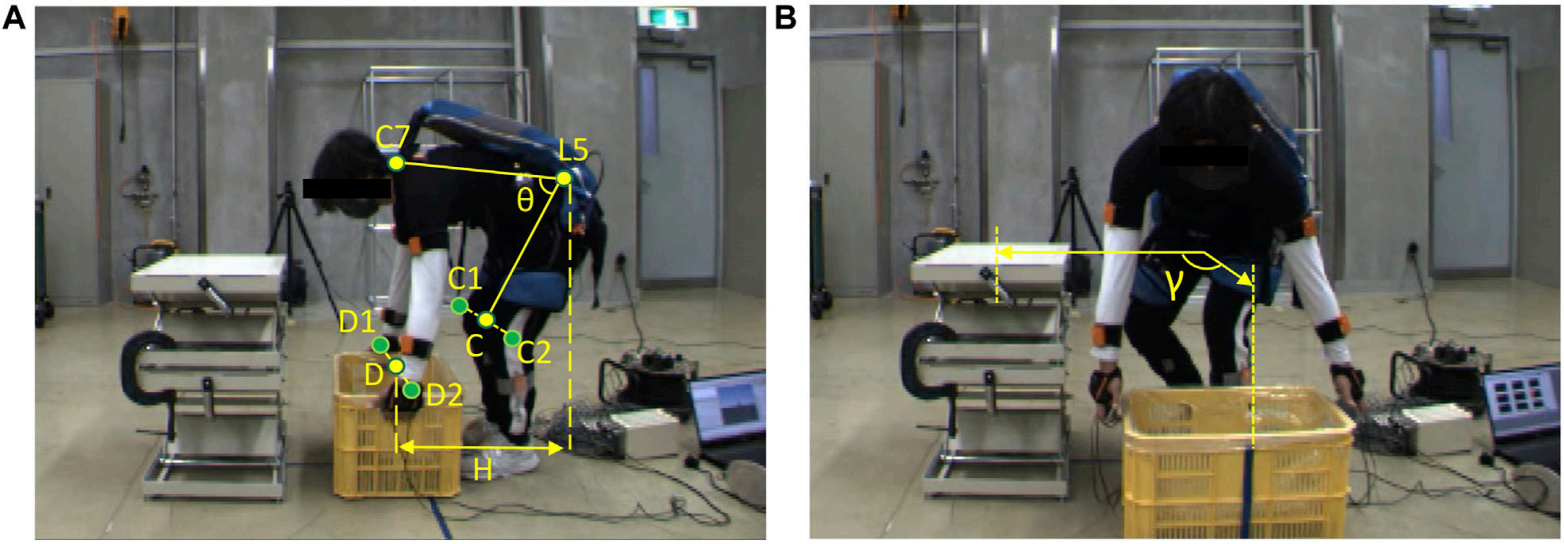
Manual handling task of a 10 kg box with an exoskeleton under **(A)** symmetrical and **(B)** asymmetrical conditions.

**FIGURE 2:**
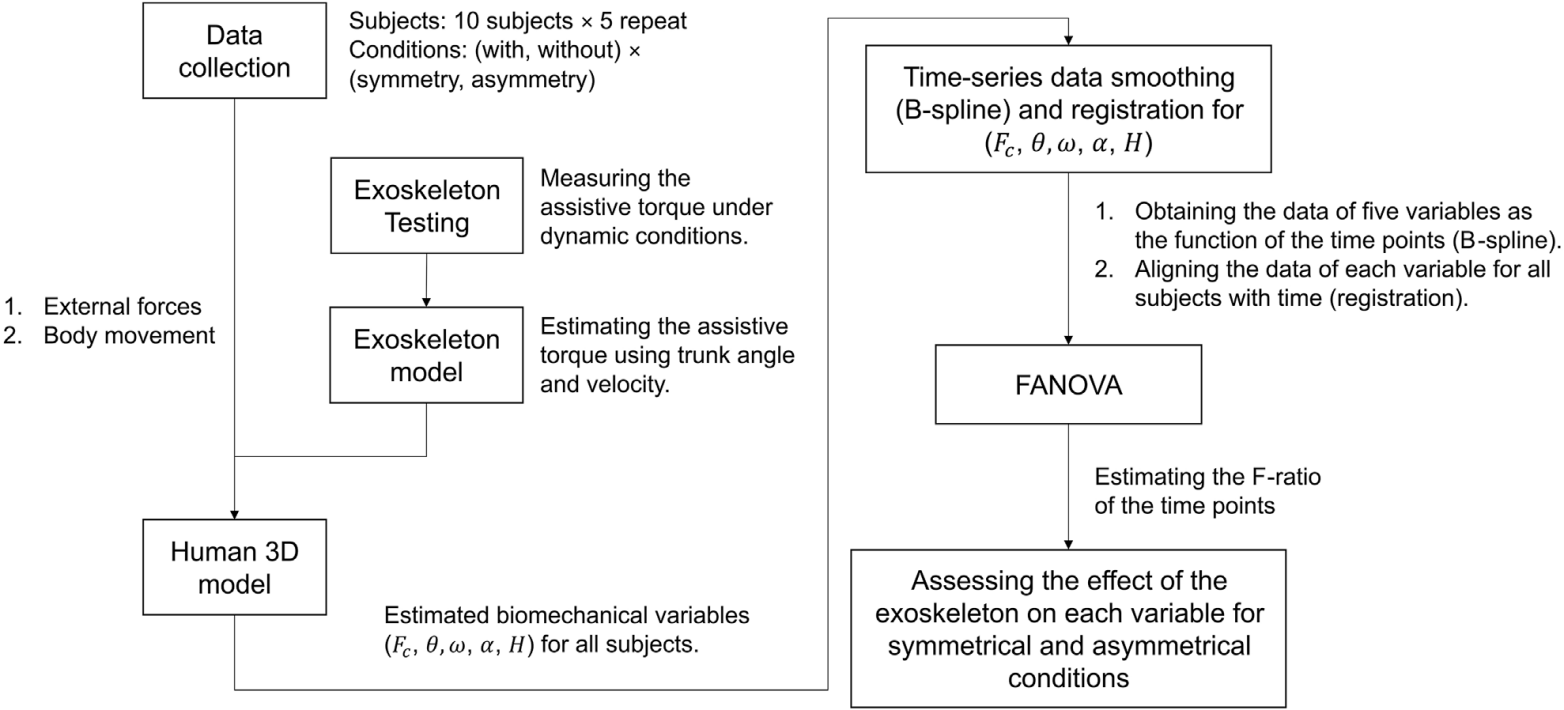
The procedure for assessing the effectiveness of the exoskeleton using FANOVA; the movement data are imported into an exoskeleton-human model to estimate the biomechanical variables, which are defined in Section 2.3; then smoothing and registration ensures the data can be presented as functions and aligned with each other; finally, conducting FANOVA and estimate F-ratio to assess the exoskeleton’s effect at the time points.

In this study, we used the FANOVA to investigate the effect of the exoskeleton. In this section, 2.1 and 2.2 show the tasks (Figure 1), and instrumentation of this experiment. In the assessment of the exoskeleton, the details are developed as two steps: estimation of biomechanical variables (Section 2.3 and Section 2.4) and FANOVA assessment (Section 2.5 and Section 2.6). As shown in Figure 2, in the first step, using a 3D human model and exoskeleton model to estimate the biomechanical variables, which will be analyzed in the second step using FANOVA after data smoothing and registration, and finally, the effect of the exoskeleton on each variable can be assessed using F-ratio of time points.

### 2.1 Participants and tasks

Ten male participants (height: 1.72 ± 0.08 m, body mass: 68.1 ± 8.8 kg, age: 30.9 ± 7.7 years) were recruited and all of them consented to join this experiment. This study was approved by the institutional review board of the Institute of Agricultural Machinery, National Agriculture and Food Research Organization (approval no. Kakushin-ken_Rinri_R03-02). Participants aged 20–40 years were selected because manual handling tasks pose a high risk of low back pain, and young individuals can tolerate relatively high lumbar loads (Kudo et al., 2019).

The manual handling tasks comprised three phases: free-flexion, box-lifting, and box-laying. Figures 1A, B show the beginning of symmetrical and asymmetrical lifting, respectively. The participants performed 2 × 2 non-repetitive tasks (asymmetrical/symmetrical condition with/without the exoskeleton) five times each, the time interval between two trials was around 30 s to reduce the effect of muscle fatigue. The participants’ feet were always pointing forward. Prior to the experiment, participants were instructed to perform manual handling tasks at their preferred speed to test their strength.

### 2.2 Instrumentation

A motion capture system (Xsens MVN Analyze, Xsens, Inc., Enschede, Netherlands) was used to reconstruct motion using IMUs. As shown in Figure 1, 15 IMUs were attached to the participants’ heads, shoulders, L5/S1, upper arms, forearms, thighs, shanks, and feet. Two identical force-measuring devices recorded external forces on the hands, each consisting of two three-axis force sensors (USL08-H6, Tec Gihan Co., Ltd., Kyoto, Japan). The box size was 57 cm × 28 cm × 10 cm, with a total mass of 10 kg, which is the limit mass for a normal adult in a one-time lift (ISO 11228-1, 2021). Data recorded at 60 Hz were filtered using a low-pass filter with a 4 Hz cut-off frequency.

### 2.3 Exoskeleton’s effect on biomechanical variables

Lumbar load reduction is the biomechanical criterion for relieving the lumbar burden (Waters et al., 1993). The kinematics variables such as trunk angle, trunk angular velocity, trunk angular acceleration, and the horizontal displacement between the wrist and the lumbar represent the effect of the exoskeleton on the motion restrictions (Potvin, 1997; Marras et al., 2000).

The five representative biomechanical variables are presented as lumbar load ( $F_{c}$ ), trunk angle ( θ ), trunk angular velocity ( ω ), trunk angular acceleration ( α ), and horizontal displacement between the wrist and lumbar ( H ). The trunk angle θ = ∠C7·L5·C is shown in Figure 1A, and the trunk angular velocity and acceleration are the derivatives of the trunk angle and angular velocity, respectively. In Figure 1A, the C7 and L5 positions are determined by virtual markers generated by the motion reconstructed using Xsens MVN Analyze. The wrist (D) and knee (C) positions were determined on the basis of the center of the virtual markers of the left and right wrist (D1, D2), as well as the left and right knee (C1, C2), respectively. During the asymmetrical task, the angle (γ) between the box and the table (65 cm height) in the horizontal plane was 90°, as shown in Figure 1B.

### 2.4 Lumbar load estimation

#### 2.4.1 Exoskeleton model

The “Muscle Suit Every” (Innophys, Inc., Tokyo, Japan) exoskeleton was used in this study. This exoskeleton uses artificial muscle to provide assistive torque at different trunk angles. In addition, angular velocity influences assistive torque by altering artificial muscle extension speed, which affects the friction force of the artificial muscle and assistive torque generation (Sugimoto et al., 2011; Tondu, 2012). Thus, we used the thin-plate spline (TPS) model for estimating assistive torque based on trunk angle and angular velocity. TPS can provide a robust estimation for spatial data interpolation and surface fitting (Bookstein, 1989; Donato Belongie, 2002).

**FIGURE 3:**
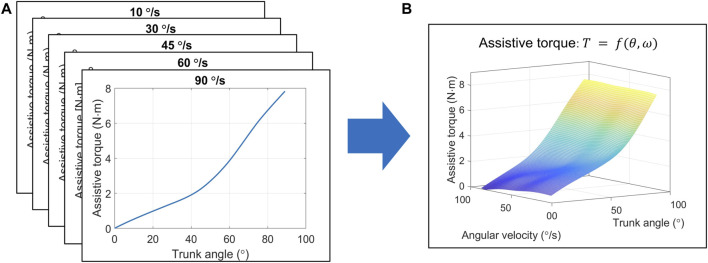
Procedure to estimate exoskeleton’s assistive torque based on the TPS method. **(A)** The measured assistive torque and trunk angle at different speeds. **(B)** The TPS model estimation of assistive torque using trunk angle and angular velocity. Abbreviations: TPS, thin-plate spline.

To establish the TPS model, we used a testing machine to record the assistive torque of the exoskeleton when the trunk angle rotated from 0° to 90° at five speeds (10, 30, 45, 60, and 90°/s) at a sampling rate of 1 kHz (Tanaka et al., 2020). In every test, air pressure of the exoskeleton was set to 0.1 MPa. The tests were repeated at each speed 10 times. Subsequently, the recorded data were processed with a low-pass filter of 10 Hz. The results, shown in Figure 3A, are the relationships between the extension angle and average assistive torque in the 10 trials under each speed condition. Finally, utilizing the TPS method, which was established using MATLAB (version 2022a), the estimated assistive torque could be presented in terms of trunk angle and angular velocity, as shown in Figure 3B. The TPS model’s R2 was 0.955 with a root mean square error of 3.70 for all testing data.

#### 2.4.2 Actual lumbar load estimation

**FIGURE 4:**
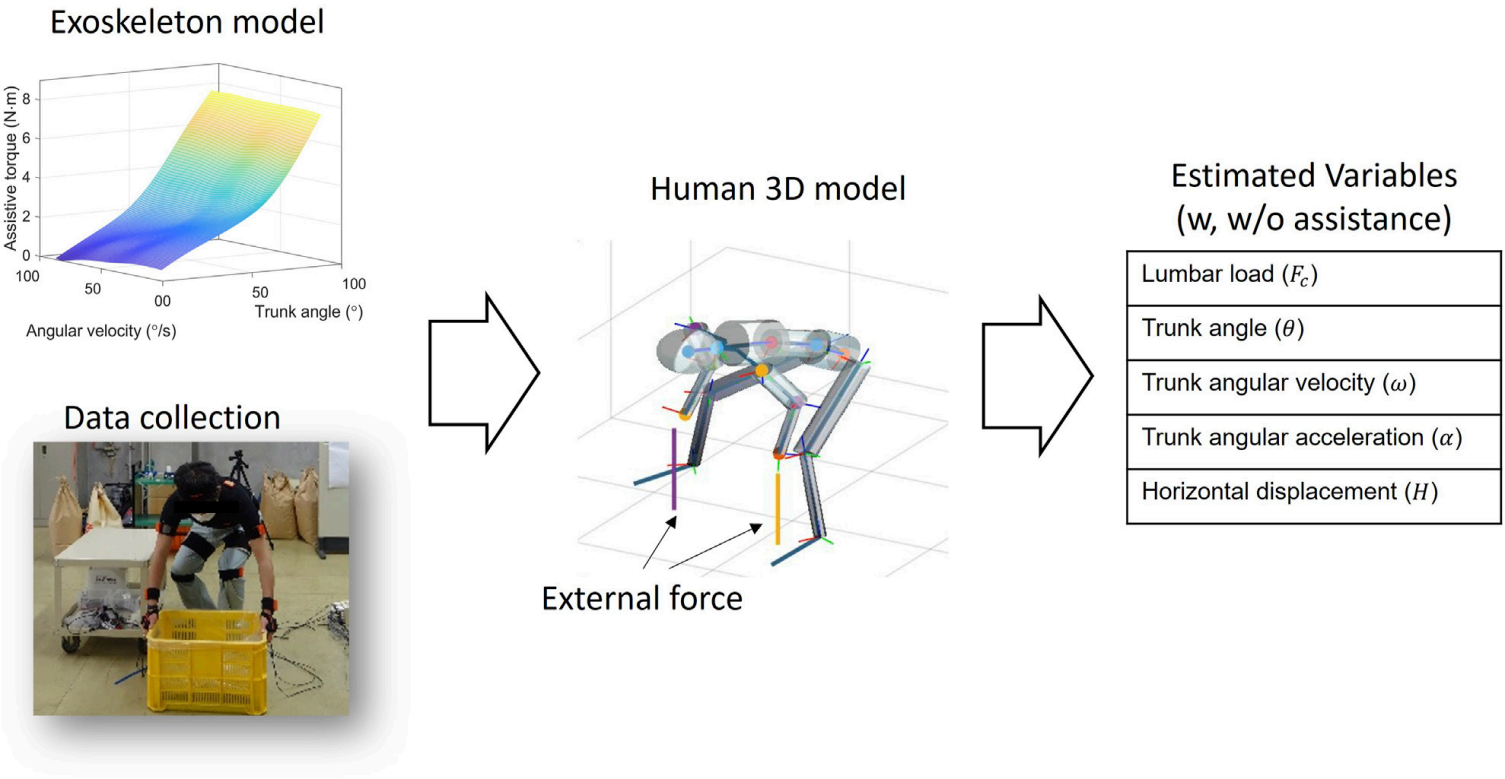
Schematic of estimating biomechanical variables using the human 3D model. After obtaining the exoskeleton model, the motion and the external force data are imported into the human 3D model, and then the biomechanical variables are estimated.

As shown in Figure 4, A link-chain human three-dimensional (3D) model was established. The coordinate, movement and center of mass for each segment, and motion reconstruction were recorded using the IMU motion capture system attached to the subject body (data collection). The dynamic link-chain model had nine segments, involving the forearms, upper arms, head, shoulder, thoracic spine, lumbar spine, and pelvis (the lower limbs are not included in the dynamic calculation). The mass of each segment was a proportion of the total body mass, as estimated by a previous study (Winter et al., 2009; Hof, 1992). In addition, the mass of the exoskeleton was added to the center mass at the lumbar segment.

To calculate the lumbar torque, we initially estimated the non-assisted lumbar torque using the top–down inverse dynamic method based on the link-chain human model. Given that the assistive torque only acts in the flexion plane (plane C7L5C in Figure 1A), we subtracted the assistive torque in this plane from the non-assisted torque to obtain the actual torque at the lumbar joint. The geometric model of the trunk muscles was determined using previously reported data (Schultz et al., 1982; Granata and Marras, 1993; Gagnon et al., 2001), and the muscular forces were estimated by minimizing the sum of the trunk muscles’ stress square (Anderson and Pandy, 2001). Finally, the lumbar load was obtained by the force resulting from the muscular forces and the upper body load in the direction perpendicular to the lumbar vertebra. The inverse dynamic computation was completed in MATLAB (version 2022a), and the optimization procedure was completed by the quadratic programming algorithm (Stellato et al., 2020).

In order to investigate the accuracy of the 3D human model, we compared the estimated lumbar load from this 3D human model with the *in vivo* data reported by Wilke (Wilke et al., 1999; Wilke et al., 2001) under several body conditions. The estimated lumbar load for each posture was obtained from the average estimation among eight volunteers with a mean body height of 1.72 m and mean body mass of 69 kg, similar to the participant (1.68 m, 70 kg) in Wilke’s study.

**FIGURE 5:**
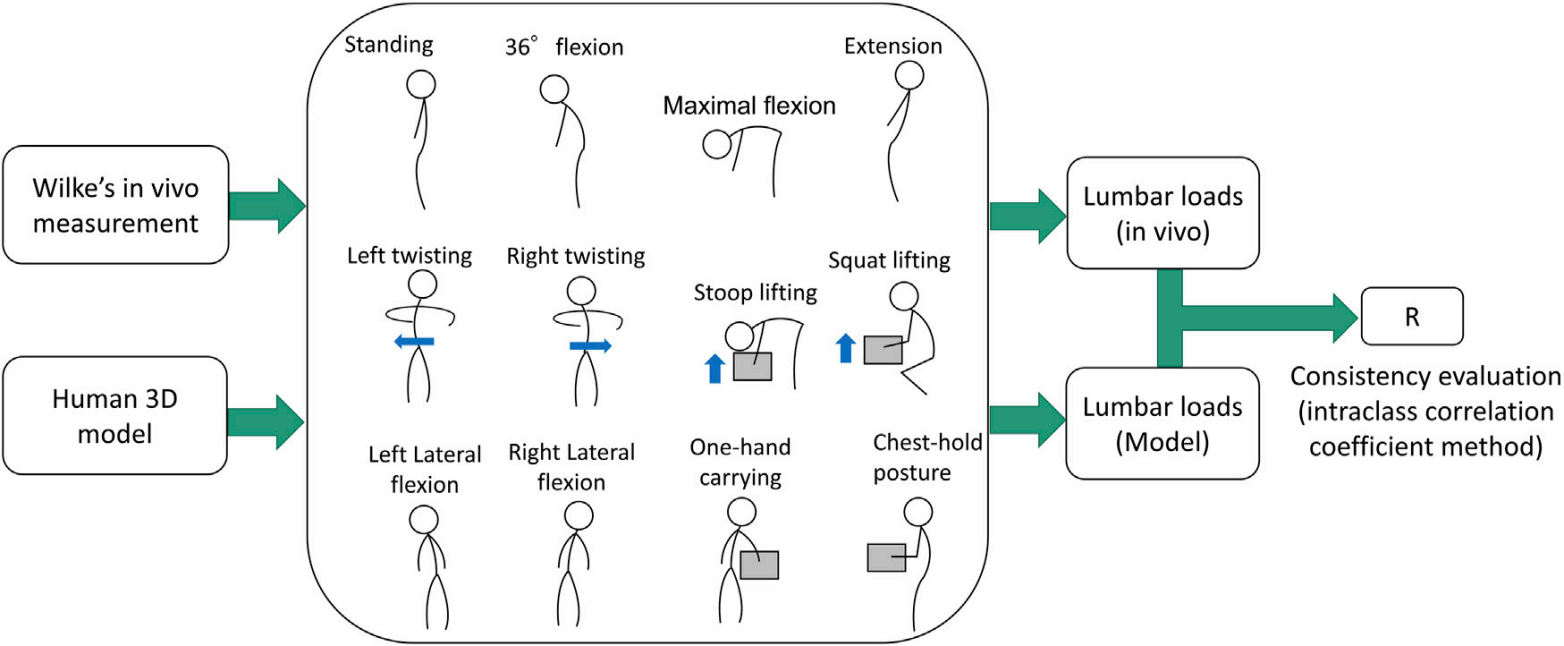
Comparison between reported the *in vivo* data and the estimated lumbar load using the 3D human model. The mass of the box is simulated as 20 kg.

The accuracy estimation procedure was presented in Figure 5, where the lumbar loads estimated by the human model at 12 postures including standing, 36° flexion, maximal flexion, extension, lateral flexion, twisting, stoop lifting, squat lifting, one-hand carrying, and close-to-chest handling were compared with these obtained from the reported *in vivo* experiments. The *in vivo* lumbar intradiscal pressure (MPa) estimated by Wilke was converted to lumbar load (N) using the correction factor proposed by Dreischarf et al. (2013). Finally, calculating the R of Intraclass Correlation Coefficient (ICC) between the estimated lumbar loads of the human model and the invasive data for the postures [type of ICC (1,1), and on the 95% confidence interval]. The estimated R is 0.93, which can be explained as excellent reliability (>0.9) (Koo and Li, 2016). The result indicates that the lumbar load estimated by the human model has a high consistency with the *in vivo* data.

### 2.5 Functional data analysis

#### 2.5.1 Smoothing and processing

This study’s biomechanical time-series discrete data, including tracks, angles, and lumbar moment, were converted to functional data in the FDA. In this step, we use a series of the basis functions to fit the recorded biomechanical data and one advantage of this step is to reduce the influence of noise. Since non-repetitive tasks were conducted in this experiment, B-spline is used as the basis function system. Each obtained function can be presented as follows (Ramsay and Silverman, 2005):

$$x_{i}\left(t\right)=\sum_{k=1}^{K}c_{ik}\varphi_{k}\left(t\right)$$ (1)

where $x_{i}\left(t\right)$ represents the function converted from the *i*-th observed data series; *t* represents the number of time points; $c_{ik}$ represents the coefficients; and $\varphi_{k}\left(t\right)$ are B-spline basis functions with the number, *K*.

The B-spline fitting functions for the recorded data can be determined using the least square method. The residual sum of squares and a penalty term based on the second derivative of the fitted curve were minimized. The following equation expresses the minimized penalized least squares problem (Ramsay and Siverman, 2005):

$$G=\sum_{i=1}^{N}{\left[X\left(t_{i}\right)-x\left(t_{i}\right)\right]}^{2}+\lambda \int_{a}^{b}{\left[D^{2}x\left(t\right)\right]}^{2}dt$$ (2)

where $X\left(t_{i}\right)$ represents the observed data points at the *i*-th time point (i = 1, 2,…, N); $x\left(t_{i}\right)$ represents the estimated data at the *i*-th time point using the function obtained with Eq. 1; λ is the smoothing variable, which is a non-negative constant that controls the smoothness of the function, with a larger value leading to a smoother function; and $D^{2}x\left(t\right)$ represents the second derivative of the fitted function in time-series. The first part measures goodness of fit between the data points and the fitted function, and its objective is to minimize the residuals. The second part is the penalty term that encourages smoothness in the fitted function by penalizing abrupt changes in curvature; the penalty term is proportional to the integral of the squared second derivative of the function $x\left(t\right)$ over the domain (a, b). Thus, this objective function allows us to control the trade-off between the goodness of fit and the smoothness of the function.

Data registration is a technique that aligns generated functional data that might be misaligned. This can improve the ANOVA before estimating the main effects and interactions. We applied data registration to all observed data for each condition and variable using continuous registration (Ramsay and Silverman, 2005).

#### 2.5.2 FANOVA

After obtaining the functional data, we performed FANOVA to investigate the effect of exoskeletons on each variable under symmetrical and asymmetrical conditions. The model for each variable, $f\left(t\right)$ , with time history can be presented as follows:

$$f\left(t\right)=\mu \left(t\right)+\alpha_{i}\left(t\right)+\varepsilon \left(t\right)$$ (3)

where $\mu \left(t\right)$ is the mean function indicating the average value of all trials under the symmetrical or asymmetrical condition; $\alpha_{i}\left(t\right)$ represents the effect of using (i = 1) and not (i = 2) exoskeletons on the variable; and $\varepsilon \left(t\right)$ is the unexplained variation. We identified the specific effects of using exoskeletons; the constraint added for all t, as $\alpha_{1}\left(t\right)+\alpha_{2}\left(t\right)=0$ .

Then, the model for each variable for each *t* can be rewritten as a matrix form $f\left(t\right)$ as follows:

$$f\left(t\right)=Z\beta \left(t\right)+\varepsilon \left(t\right)$$ (4)

where $f\left(t\right)$ is the 20 × 1 function vector and Z is the 20 × 3 design matrix, with the 20 rows corresponding to the 10 participants, each contributing with two curves: one when using exoskeletons, and the other when not using them. The first column has ones; the second column has zeros in the first 10 rows, followed by ones; and the final column has ones in the first 10 rows, followed by zeros. $\beta \left(t\right)$ is the 3 × 1 vector of parameter functions, with $\beta \left(t\right)={\left[\mu \left(t\right),\alpha_{1}\left(t\right),\alpha_{2}\left(t\right)\right]}^{\prime }$ ; $\varepsilon \left(t\right)$ is the 20 × 1 vector of residual functions.

The vector $\beta \left(t\right)$ can be estimated by minimizing the linear minimum mean square error (LMSSE):

$$\text{LMSSE}\left(\beta \right)=\int {\left[f\left(t\right)-Z\beta \left(t\right)\right]}^{\prime }\left[f\left(t\right)-Z\beta \left(t\right)\right]dt$$ (5)

$\text{LMSSE}\left(\beta \right)$ should be minimized under the condition $\alpha_{1}\left(t\right)+\alpha_{2}\left(t\right)=0$ .

As with traditional ANOVA, the error sum of squares for the residual function and the mean curve were evaluated as a function for each time point in (*t*) with the following. SSE (sum of squared errors) and SSY (sum of squared in a total) of the model at time *t* can be calculated as:

$$\text{SSE}\left(t\right)={\sum \left[f\left(t\right)-Z\hat{\beta }\left(t\right)\right]}^{2}$$ (6)

$$\text{SSY}\left(t\right)={\sum \left[f\left(t\right)-\hat{\mu }\left(t\right)\right]}^{2}$$ (7)

where $\hat{\beta }\left(t\right)$ is the 20 × 1 vector of the predicted parameters function; $\hat{\mu }\left(t\right)$ is the 20 × 1 vector of the predicted mean function of all trials.

The F-ratio determines whether the variance between two data sets is equal, and the FANOVA can evaluate the F-ratio across time *t*. The F-ratio can be presented as follows:

$$F–\text{ratio}\left(t\right)=\frac{\left[\text{SSY}\left(t\right)-\text{SSE}\left(t\right)\right]/\text{df}\left(\text{error}\right)}{\text{SSE}\left(t\right)/\text{df}\left(\text{regression}\right)}$$ (8)

where df(error) is the degree of freedom for error [df(error) = 1], and the df(regression) is the difference in degrees of freedom [df(regression) = 18]. Based on the F-criterion is 4.41. Any time the functional curve F-ratio > F-criterion, the effect of time had reached significance at the chosen *α* level of 0.05.

### 2.6 Statistical analysis

For all tasks, the paired *t*-test (at an *α* level of 0.05) was used to compare the peak values of the lumbar load, trunk angle, angular velocity, angular acceleration, and horizontal distance between the wrist and lumbar spine under symmetrical and asymmetrical conditions. Subsequently, the result of *t*-test was compared to that of FANOVA, which was utilized to examine the continuous effect of the exoskeletons on these variables throughout the normalized manual handling tasks. The F-ratio, obtained as time history, was compared to the F-criterion at an *α* level of 0.05.

The functional analysis for time-series data was performed using the package developed by Ramsey and Silverman, available at https://www.psych.mcgill.ca/misc/fda/downloads/FDAfuns/. All the statistical analyses were conducted using MATLAB (version 2022a).

## 3 Results

### 3.1 T-test analysis: effect on peak values of each variable

**FIGURE 6:**
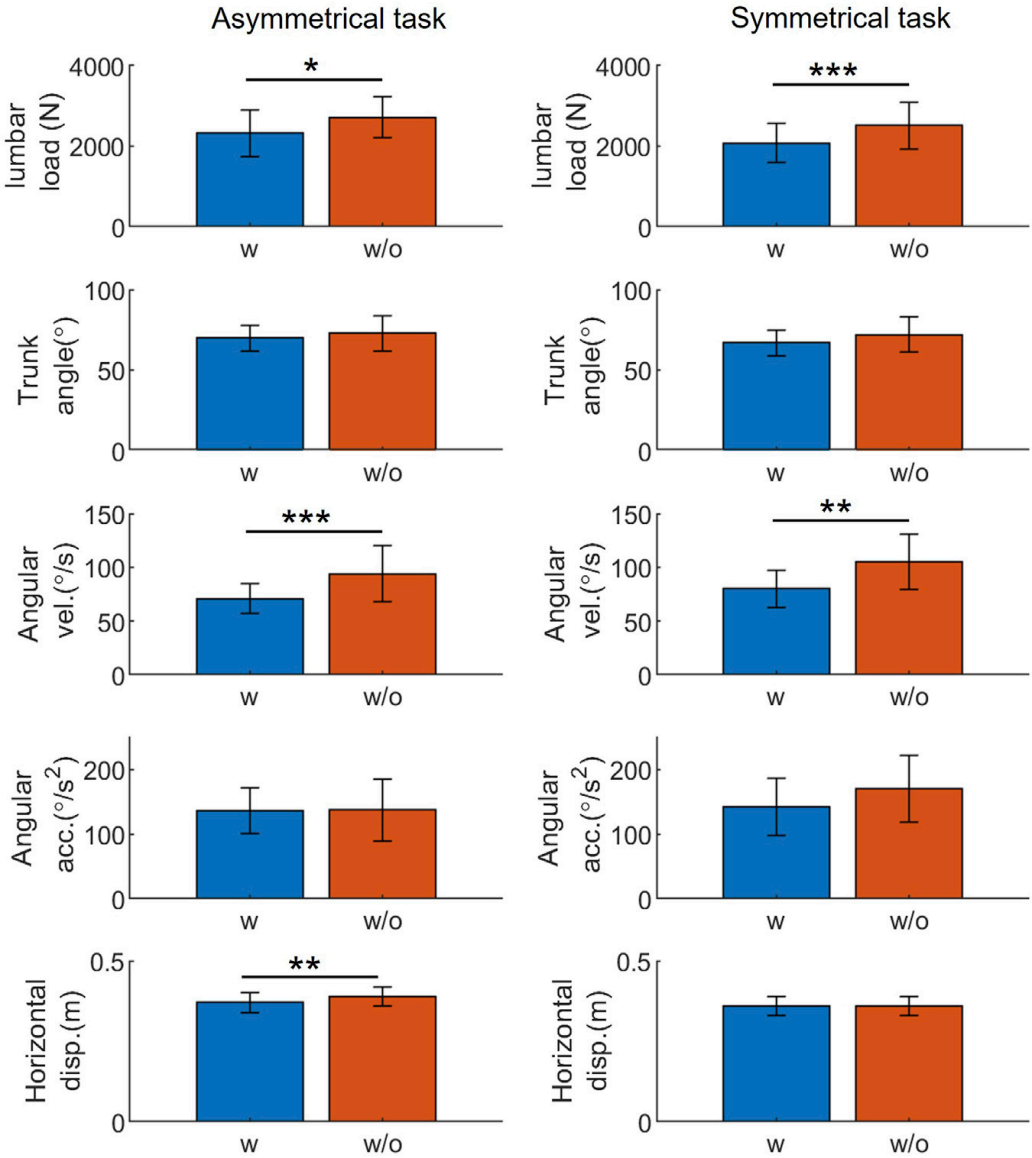
The *t*-test result: exoskeletons’ influence on the peak value of representative variables during manual handling tasks. Abbreviations: acc., acceleration; disp., displacement; vel., velocity; w, with; w/o, without (*p < 0.05; **p < 0.01; ***p < 0.005).

The estimated peak variables of lumbar load, trunk angle, angular velocity, angular acceleration, and horizontal displacement between the wrist and lumbar are shown in Figure 6, which shows the average peak variables and standard deviations for all participants. Peak lumbar load and angular velocity were significantly lower (*p* < 0.05) when using the exoskeleton during both tasks; however, peak horizontal displacement was significantly lower only during asymmetrical tasks (*p* < 0.01). Compared with not using exoskeletons, using them reduced peak lumbar load by 388 N (14%) and 427 N (17%) during both tasks, respectively. Similarly, the peak trunk angular velocity reduction was 23°/s (24%) and 25°/s (24%) under both conditions. During asymmetrical tasks, the peak horizontal displacement was reduced by 0.02 m (5%). No significant difference was observed in peak trunk angles and angular acceleration variables for both tasks.

### 3.2 FANOVA: effect on time-series values of each variable

**FIGURE 7:**
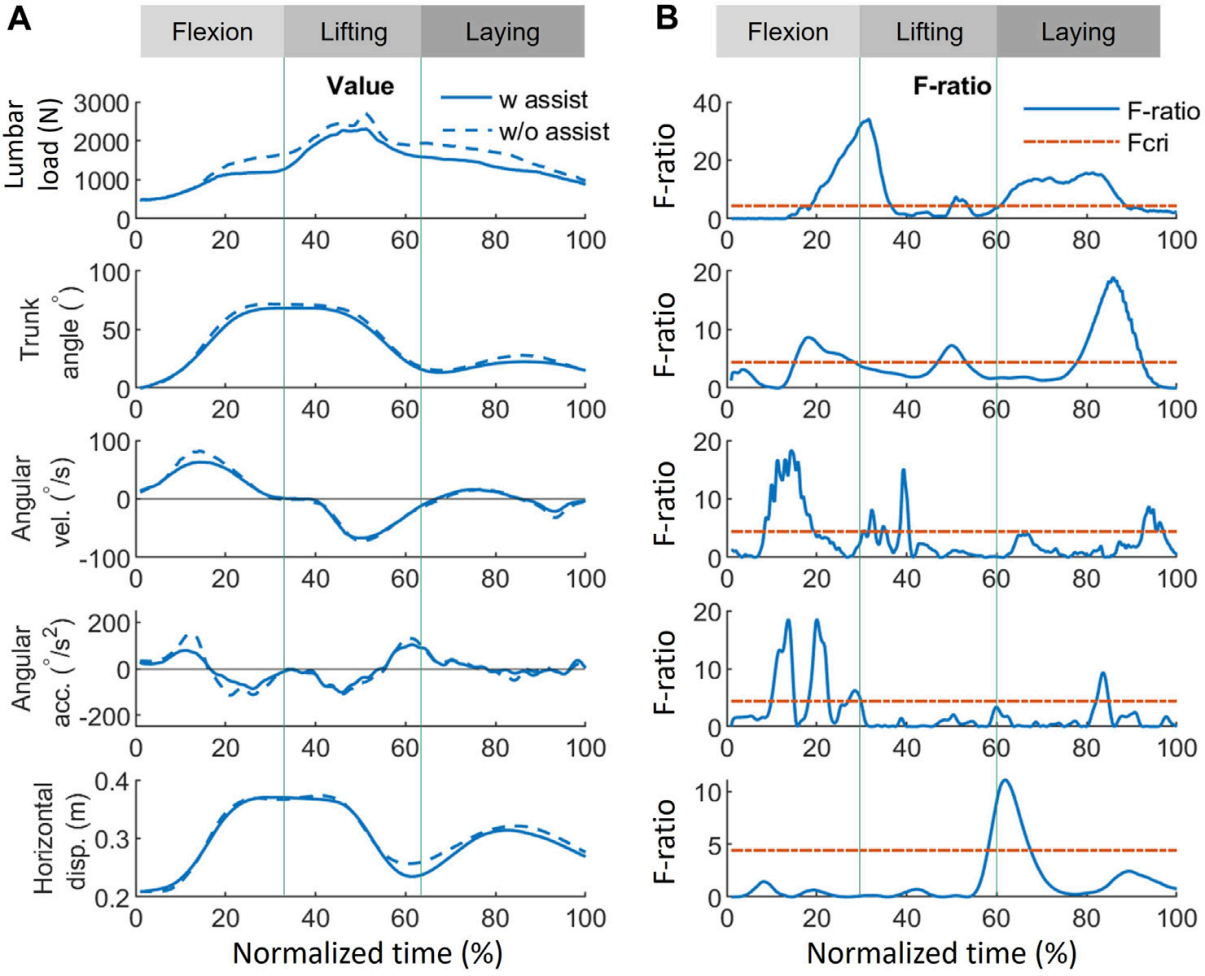
Effect of using exoskeletons on the variables during asymmetrical tasks. **(A)** Absolute values of the variables when using (solid line) and not using (dash line) exoskeletons, where positive (+) and negative (−) for angular velocity and acceleration represent flexion and lifting, respectively. **(B)** The corresponding F-ratio generated by FANOVA (solid line) and Fcri = 4.41 with statistical significance at an *α* level of 0.05 (red dash line). The whole normalized time is separated into flexion (0%–33%), lifting (33%–67%), and laying (67%–100%) phases. Abbreviations: FANOVA, functional analysis of variance; Fcri, F-criterion; acc., acceleration; disp., displacement; vel., velocity; w, with; w/o, without.

**FIGURE 8:**
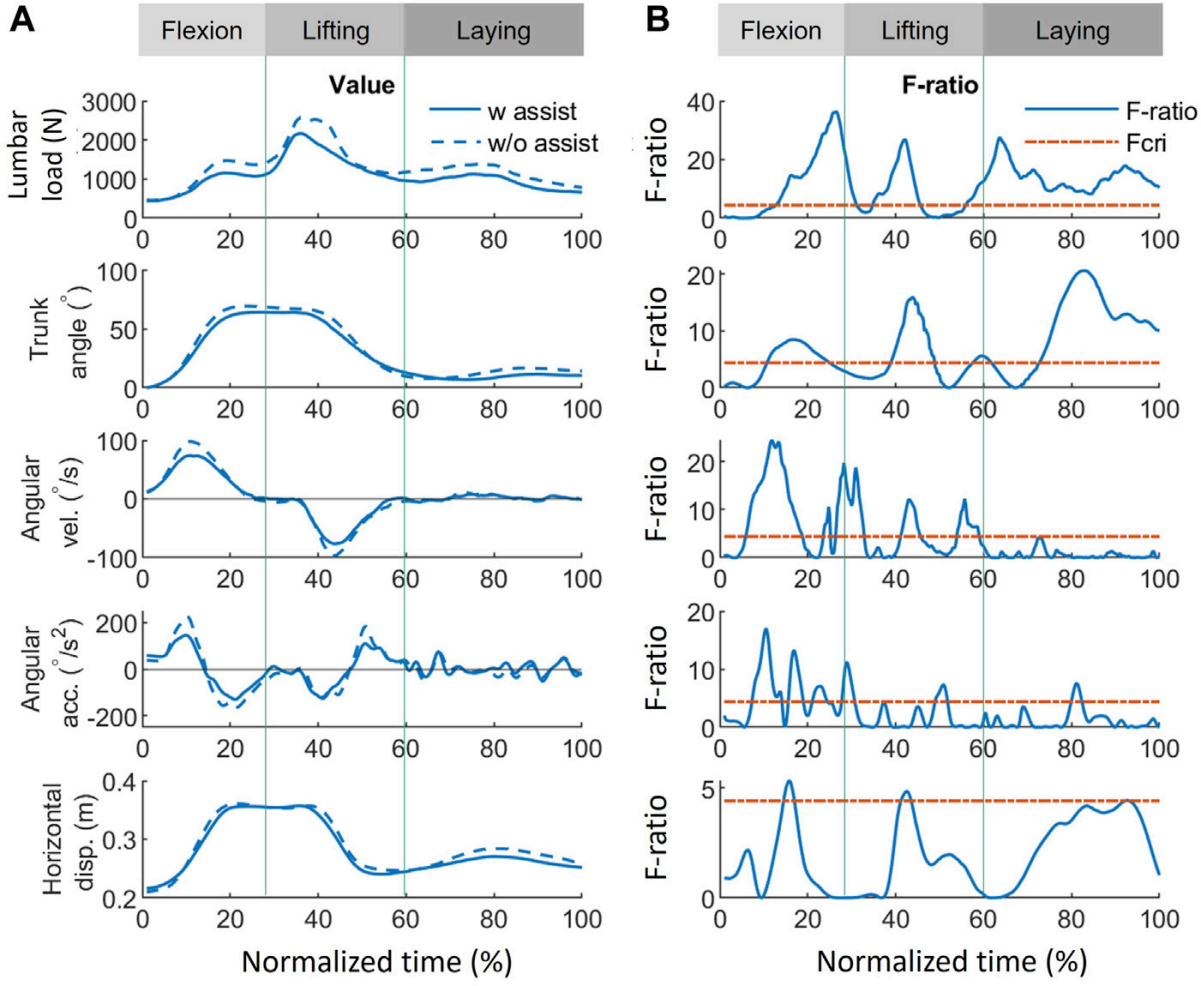
Effect of using exoskeletons on the variables during symmetrical tasks. **(A)** Absolute values of the variables when using (solid line) and not using (dash line) exoskeletons, where positive (+) and negative (−) for angular velocity and acceleration represent flexion and lifting, respectively. **(B)** The corresponding F-ratio obtained by FANOVA (solid line) and Fcri = 4.41 (red dash line). The whole normalized time is separated into flexion (0%–30%), lifting (30%–60%), and laying (60%–100%) phases. Abbreviations: FANOVA, functional analysis of variance; Fcri, F-criterion; acc., acceleration; disp., displacement; vel., velocity; w, with; w/o, without.

**FIGURE 9:**
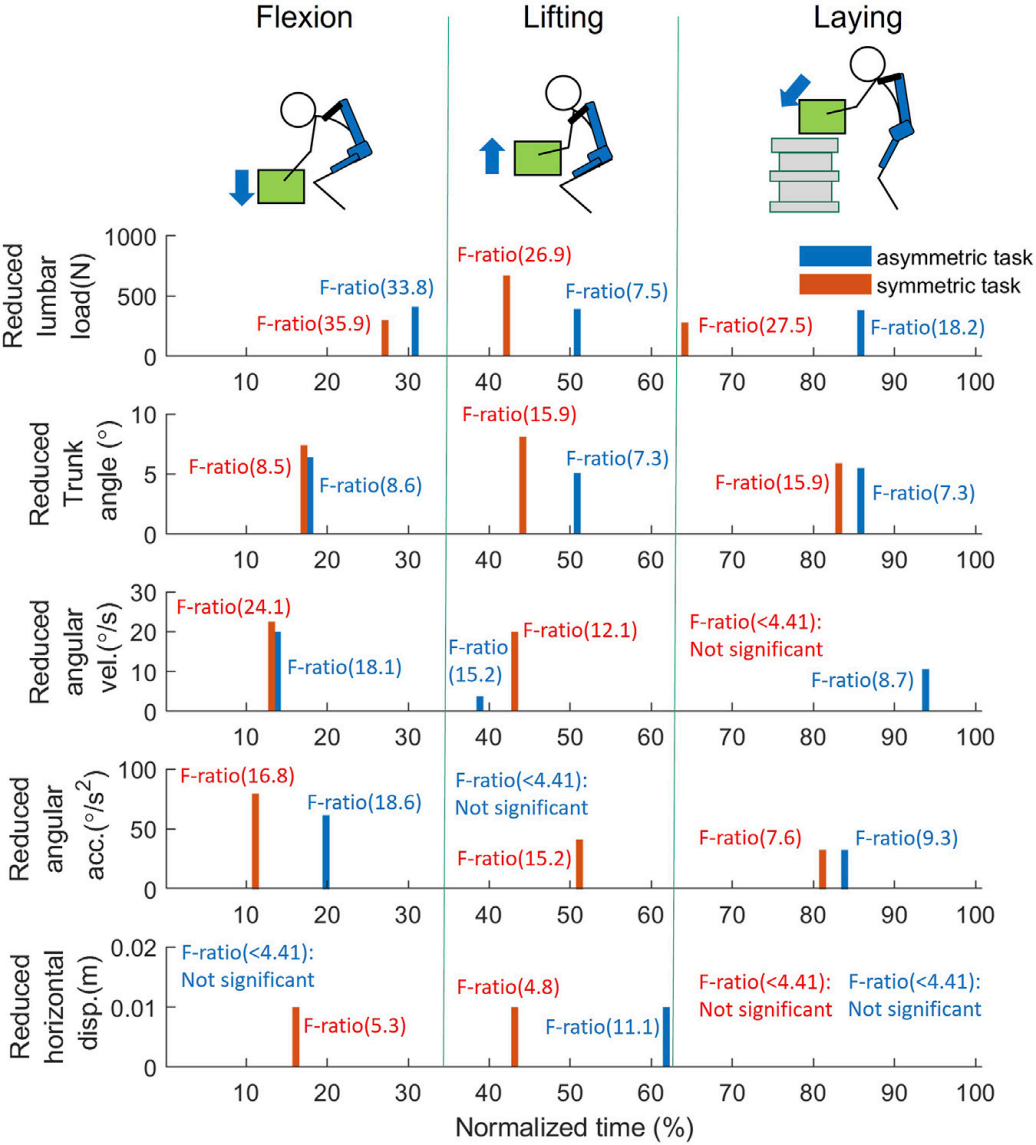
FANOVA result: The reduced variables at the peak F-ratio timing for each phase during the manual handling task between w, and w/o assistance; The bar values, representing the reduction of each variable (blue: asymmetrical task; red: symmetrical task), and are obtained at the peak F-ratio of flexion, lifting, and laying phase, respectively. As F-ratio is smaller than 4.41, it is considered no significant difference is found between w, and w/o assistance. Abbreviations: acc., acceleration; disp., displacement; vel., velocity.

The results of the functional analysis, as presented in Figures 7–9, demonstrate the effect of using exoskeletons on different variables during asymmetrical and symmetrical tasks.

During asymmetrical tasks (Figure 7), the exoskeleton reduced lumbar load by 412, 393, and 383 N with the greatest significant differences during the flexion, lifting, and laying phases, respectively. In addition, the most significant lumbar load reduction (412 N, F-ratio = 33.8) for all phases occurred during the flexion phase (31.7%), as shown in Figure 9. Reductions in the kinematic variables of trunk angle, angular velocity, and angular acceleration were observed in all phases (Figure 7), indicating the exoskeletons’ impact on posture and movement restrictions during asymmetrical tasks. As shown in Figure 9, the most significant reduction (5.5°, F-ratio = 18.6) in trunk angle occurred during the laying phase (86.1%); for trunk angular velocity, the reduction (20.0°/s, F-ratio = 18.1) occurred during the flexion phase (14.1%); and for trunk angular acceleration, the reduction (61.5°/s^2^, F-ratio = 18.6) occurred during the flexion phase (20.0%). Horizontal displacement was only significantly reduced during the lifting phase (61.9%), by 0.02 m (F-ratio = 11.1).

During symmetrical tasks (Figure 8), the exoskeleton reduced lumbar load by 300, 672, and 280 N with the greatest significant differences during the flexion, lifting, and laying phases, respectively. The most significant lumbar load reduction (300 N, F-ratio = 35.9) for all phases occurred during the flexion phase (26.7%) (Figure 9). Similarly, in asymmetrical tasks, reductions in the kinematic variables such as trunk angle, angular velocity, and angular acceleration were observed in all phases (Figure 8). As shown in Figure 9, the most significant reduction in trunk angle (5.9°, F-ratio = 20.6) occurred during the laying phase (83.2%) and that in trunk angular velocity (22.5°/s, F-ratio = 24.1) during the flexion phase (13.4%). For trunk angular acceleration, the reduction (79.4°/s^2^, F-ratio = 16.8) occurred during the flexion phase (10.6%). The patterns of the above kinematic variables were also similar to those in asymmetrical tasks. Horizontal displacement was significantly reduced during the lifting (15.8%) and flexion (42.6%) phases by 0.02 m with similar F-ratios (5.3 vs. 4.8).

Except for providing assistive torque, exoskeletons can reduce the lumbar load by restricting movements. As shown in Figures 7, 8, some significant overlaps were observed between the F-ratio of the lumbar load and the kinematic variables in each phase. During the flexion phase, these overlaps of the significant F-ratios were as follows: lumbar load: 13%–31%; trunk angle: 13%–24%; angular velocity: 13%–18%, 25%–31% (in the flexion phase, both periods were where F-ratios of angular velocity were significant); angular acceleration: 15%–30%; and horizontal displacement: 14%–17%. During the lifting phase, lumbar load: 35%–45%; trunk angle: 39%–49%; angular velocity: 41%–45%; and horizontal displacement: 41%–43%. During the laying phase, lumbar load: 56%–100%; trunk angle: 73%–100%; angular velocity: 56%–58%; and angular acceleration: 79%–82%. These overlaps show the consistency between the reduction of lumbar load and that of kinematic variables, which indicate that the lumbar load reduction is not only affected by lumbar moment reduction but also by restricting the kinematic variables when using the exoskeletons.

## 4 Discussion

### 4.1 T-test analysis: effect on peak values of each variable

The findings presented in Figure 6 show that exoskeletons significantly affect the representative biomechanical variables during symmetrical and asymmetrical tasks. Exoskeleton use substantially reduced peak lumbar load and motion speed, consistent with previous findings (Koopman et al., 2019b). Increasing trunk angular velocity requires greater trunk muscle activation (Dolan and Adams, 1993). This result aligns with those of previous studies, suggesting that exoskeletons help reduce lumbar load and muscle activation (Huysamen et al., 2018; Lamer et al., 2018), thus, lowering the risk of musculoskeletal disorders and enhancing worker comfort.

No significant differences were detected in peak trunk angles and angular accelerations, in contrast to angular velocity, for both tasks. Thus, it results difficult to infer from peak values whether exoskeletons limit the range of motion or hinder human movement while providing support and reducing lumbar load. Previous studies have yielded mixed results on the exoskeletons’ influence on trunk angle: reduction was observed on the nylon elastic support, while no reduction was shown for another passive exoskeleton (Laevo V2.4 Delft, Netherlands) (Marras et al., 2000; Koopman et al., 2019a). The differing results from our experiment and previous studies regarding the influence of exoskeleton on trunk angle can be attributed to variations in design.

The lumbar reduction of the current exoskeleton can meet the 3.4 kN average lumbar load criterion recommended by the National Institute of Occupational Safety and Health (Waters et al., 1993), as shown in Figure 6. However, the lumbar load limit will vary across age groups and sexes (Genaidy et al., 1993). Aging societies have a growing population of older workers, whose lumbar load limit is 1.69 kN lower than that of younger workers (Kudo et al., 2019). Women have lower lumbar load limits than men (Genaidy et al., 1993; Kudo et al., 2019). Thus, deterministic assistive forces should consider lumbar load limits for different age groups and sexes.

### 4.2 FANOVA: effect on time-series values of each variable

FANOVA enables a more comprehensive examination of the effects of exoskeletons on variables throughout an entire task rather than focusing solely on peak values. The FANOVA results suggested that exoskeletons can alleviate lumbar burden at peak load timings and throughout all task phases (Figure 9). The lumbar load reduction in Figures 7, 8 implies that exoskeletons are probably effective in reducing the lumbar load in all phases during asymmetrical tasks while being much more effective in the lifting phase during symmetrical tasks. Asymmetrical lifting reportedly results in a higher lumbar load than symmetrical lifting, implying that workers are more easily prone to getting lumbar injuries during asymmetrical lifting (Kim and Zhang, 2017). Therefore, improving exoskeletons’ performance during asymmetrical lifting can reduce users’ lumbar injury risks.

The reduced trunk angle, angular velocity, and acceleration during both manual handling tasks suggest a similar tendency when exoskeletons assist participants. The results indicated that using the exoskeleton imposes significant restrictions on body movement at larger flexion postures (>25°), in the vicinity of peak lumbar load occurrence, and throughout most of the box-laying movements in both tasks. The most significant reduction in angular velocity and acceleration occurred in the middle of flexion, implying that the human body may experience greater kinematic restrictions from exoskeleton use when not under loading conditions. This could be a reason for exoskeletons not supporting the lumbar load in a small trunk angle, which will be discussed subsequently. However, the restrictions on horizontal displacement differ between symmetrical and asymmetrical conditions. Under asymmetrical conditions, a significant reduction occurred at the end of lifting. In contrast, in symmetrical tasks, restrictions occurred in the middle of flexion and the vicinity of peak lumbar load occurrence. Determining the relationship between these changes using other variables in this study was challenging. This discrepancy in horizontal displacement restrictions may originate from the interaction between the trunk and upper limbs.

Comparing lumbar load and trunk angle in Figures 7, 8, significant reductions when using the exoskeletons were difficult to observe when the trunk angle was less than 25°. The only exception was during the asymmetrical lifting phase, when a significant lumbar load reduction occurred even with a trunk angle smaller than 25°. This lumbar load reduction was likely the result of reduced horizontal displacement rather than trunk angle changes. These results suggest that exoskeletons at smaller trunk angles (<25°) neither significantly reduce lumbar load nor restrict trunk movement. Since the greatest lumbar load typically occurs at larger trunk angles, this design approach may prioritize allowing users a greater range of motion and, consequently, sacrifice effectiveness at smaller trunk angles. This trade-off between range of motion and lumbar load reduction at smaller trunk angles may be a deliberate design choice to improve user comfort and task efficiency while offering lumbar support when needed.

Although restricting body movement may lead to inconvenience, adopting a low-speed or small-inclination posture will lead to lower lumbar injury risk (Marras et al., 1993; Waters et al., 1993). In addition, the lumbar load can also be reduced by decreasing the kinematic variables. The overlap of F-ratios with significance (F-ratio > 4.41) suggests that the reduction in lumbar load is affected by the kinematic variables in all phases. Significant trunk angle and horizontal displacement reductions were observed near peak lumbar load during symmetrical tasks. During asymmetrical tasks, only trunk angle reduction was noted. Quantitatively evaluating the impact of each kinematic factor on lumbar load reduction using FANOVA in conjunction with established ergonomic equations is possible (Potvin, 1997; Merryweather et al., 2009; Arjmand et al., 2011; Arjmand et al., 2012). Although quantitative assessment methods for the inconvenience of exoskeletons are lacking, the discomfort in kinematic restrictions probably results from contact pressure and friction (Baltrusch et al., 2018; Huysamen et al., 2018). Further investigation into the complex interplay between the various biomechanical variables and the different conditions could improve the understanding of the effects of exoskeleton use on human kinematics.

### 4.3 Exoskeleton simulation method

Direct measurement methods, such as electromyography or extension force, may introduce errors due to individual differences (Marras et al., 2000; Abdoli-Eramaki et al., 2007; Lamers et al., 2018). To address this, we employed a 3D interpolation method, the TPS, to represent the relationship between extension angle, angular velocity, and assistive torque. Comparing to torque–angle relationship (Koopman et al., 2019a), our approach accounts for certain dynamic factors affecting assistive torque. The advantages of TPS include efficient overfitting reduction through regularization, reduced computational complexity once the analytical form of the TPS model can be obtained, and adaptability to multidimensional datasets (Bookstein, 1989; Donato and Belongie, 2002), making it suitable for modeling various exoskeletons whose assistive torques may be influenced by different factors. Consequently, TPS is expected to contribute to exoskeleton standardization. By incorporating TPS in exoskeleton testing and refining FANOVA assessment methods, researchers can develop more effective, comfortable, and efficient exoskeletons that cater to workers’ diverse needs in various industries.

### 4.4 Comparison between FANOVA and other methods

Compared to the traditional methods such as a *t*-test or ANOVA, FANOVA includes a smoothing and data registration procedure that reduces the noise and variability of the timing/phases. Therefore, the biomechanical variables can be analyzed at each time point.

In this study, we conducted a *t*-test and determined how the peak value was affected by the dynamic movement of an exoskeleton. However, it was difficult to investigate the significant differences in the variables at the other time points because of the variability of the timing/phases of subjects. To reduce this variability, previous studies usually had subjects maintain specific postures before applying a *t*-test or ANOVA (Lamers et al., 2018; Koopman et al., 2019a). For example, Lamers et al. (2018) found that using an exoskeleton could significantly reduce a user’s lumbar load under flexion angles of 30°, 60°, and 90°. However, the static assumption may cause the lumbar load to be underestimated compared with those of actual tasks, which are usually dynamic (van Dieën et al., 2010). Moreover, it is a significant task to evaluate the effectiveness of an exoskeleton at all flexion angles, users are normally concerned about how to best use it (Upasani et al., 2019). In contrast, FANOVA makes it possible to evaluate the reduction in the dynamic lumbar load resulting from the use of an exoskeleton, and we found that the lumbar load was reduced at the majority of the flexion angles, with the exception of small angles (<25°) or during a flexion-lifting shift. Thus, compared to the traditional *t*-test and ANOVA, FANOVA can find the most effective conditions for using an exoskeleton.

FANOVA can also find the difference in the flexion angle at times other than under peak or specific conditions. Using a *t*-test, no significant difference can be found in the peak flexion angle under either a dynamic movement (Figure 6) or in static postures (Lamers et al., 2018). However, FANOVA shows that a significant restriction of the trunk angle can be found in all phases when an exoskeleton is used (Figures 7, 8). This could be because the exoskeleton mainly affects the flexion angle not at the beginning or end of lifting or flexion, but during the middle of the task.

The results of this study are expected to contribute to safety standards for exoskeletons. The safety requirements set forth in the international standard for wearable robots, ISO 13482, are limited only to conceptual design guidelines. This study investigated a method that is expected to assist manufacturers in quantitatively evaluating their products throughout the entire movement process and guide users in the appropriate use of an exoskeleton in lifting-flexion tasks.

### 4.5 Limitations

This study had a few limitations. First, this study relied on a single type of exoskeleton, although design can significantly influence an exoskeleton’s effectiveness in reducing lumbar load and modifying kinematic variables (Baltrusch et al., 2018; Kozinc et al., 2020; Luger et al., 2021). Second, the complex and time-intensive computations involved in FDA, such as the data registration procedure, may present challenges as the curves involve a considerable amount of data from lengthy experiments.

Furthermore, multiple comparisons may pose limitations. As the number of exoskeleton modes increases and significant differences are assessed at each time point, methods such as the Bonferroni adjustment, which may reduce the statistical power of the analysis, can be used (Khalaf et al., 1999). An alternative approach is establishing a critical number of simultaneous F-ratios that must exceed the F-crit to be considered significant for that period. However, the optimal number of F-ratios for this method remains unestablished (Godwin et al., 2010).

Other than the criteria mentioned, cumulative load can also contribute to low back pain. However, the lack of a safety criterion for cumulative load makes evaluating the safety of exoskeletons’ assistance on this variable difficult, and the value of the safety criterion will affect the evaluation of the exoskeletons’ assistance.

This experiment took an interval of around 30 s between two tasks. However, considering individual differences, taking a maximal voluntary contraction (MVC) test after resting would be a better way to confirm whether the rest time was sufficient to reduce muscle fatigue, which was a limitation of our work.

Despite these limitations, the study demonstrates the potential of using FANOVA to assess the effectiveness of exoskeletons in various manual handling tasks. Future research could focus on refining measurement techniques, developing more accessible tools for FANOVA, and exploring alternative statistical approaches for handling multiple comparisons.

## 5 Conclusion

A dynamic assessment method based on FANOVA was used to investigate the effect of utilizing exoskeletons on five representative biomechanical variables. The result implied that exoskeletons could reduce the lumbar load during manual handling tasks, particularly under symmetrical lifting conditions. The significant reductions in lumbar load and kinematic variables indicate that exoskeletons are crucial in protecting users’ lumbar spine and reducing the risk of low back injury. Furthermore, the exoskeletons achieve this reduction by restricting movement, which helps to maintain proper posture during handling tasks. The results also showed how exoskeletons indirectly affect the lumbar load, influencing other kinematic variables in time history. These findings contribute to developing safer and more effective exoskeleton designs, ultimately enhancing the practical adoption of exoskeletons in various scenarios involving manual handling tasks such as in agriculture, industry, and physical rehabilitation.

## Data Availability

The raw data supporting the conclusion of this article will be made available by the authors, without undue reservation.

## References

[B1] Abdoli-E M. Stevenson J. M. (2008). The effect of on-body lift assistive device on the lumbar 3D dynamic moments and EMG during asymmetric freestyle lifting. *Clin. Biomech.* (Bristol, Avon) 23 (3), 372–380. 10.1016/j.clinbiomech.2007.10.012 18093709

[B2] Abdoli-Eramaki M. Stevenson J. M. Reid S. A. Bryant T. J. (2007). Mathematical and empirical proof of principle for an on-body personal lift augmentation device (PLAD). J. Biomech. 40 (8), 1694–1700. 10.1016/j.jbiomech.2006.09.006 17466313

[B3] Ajoudani A. Zanchettin A. M. Ivaldi S. Albu-Schäffer A. Kosuge K. Khatib O. (2018). Progress and prospects of the human–robot collaboration. Aut. Robots 42, 957–975. 10.1007/s10514-017-9677-2

[B4] Anderson F. C. Pandy M. G. (2001). Static and dynamic optimization solutions for gait are practically equivalent. J. Biomech. 34 (2), 153–161. 10.1016/s0021-9290(00)00155-x 11165278

[B5] Arjmand N. Plamondon A. Shirazi-Adl A. Larivière C. Parnianpour M. (2011). Predictive equations to estimate spinal loads in symmetric lifting tasks. J. Biomech. 44 (1), 84–91. 10.1016/j.jbiomech.2010.08.028 20850750

[B6] Arjmand N. Plamondon A. Shirazi-Adl A. Parnianpour M. Larivière C. (2012). Predictive equations for lumbar spine loads in load-dependent asymmetric one- and two-handed lifting activities. *Clin. Biomech.* (Bristol, Avon) 27 (6), 537–544. 10.1016/j.clinbiomech.2011.12.015 22265249

[B7] Baltrusch S. J. van Dieën J. H. van Bennekom C. A. M. Houdijk H. (2018). The effect of a passive trunk exoskeleton on functional performance in healthy individuals. Appl. Ergon. 72, 94–106. 10.1016/j.apergo.2018.04.007 29885731

[B8] Bookstein F. L. (1989). Principal warps: thin-plate splines and the decomposition of deformations. IEEE Trans. Pattern Anal. Mach. Intell. 11 (6), 567–585. 10.1109/34.24792

[B9] De Looze M. P. Bosch T. Krause F. Stadler K. S. O’Sullivan L. W. (2016). Exoskeletons for industrial application and their potential effects on physical work load. Ergonomics 59 (5), 671–681. 10.1080/00140139.2015.1081988 26444053

[B10] Dolan P. Adams M. A. (1993). The relationship between EMG activity and extensor moment generation in the erector spinae muscles during bending and lifting activities. J. Biomech. 26 (4–5), 513–522. 10.1016/0021-9290(93)90013-5 8478353

[B11] Donato G. Belongie S. (2002). Approximate thin plate spline mappings. Comput. Vision—ECCV 2002. Proc. Part Iii 7th Eur. Conf. Comput. Vis. Cph. 7, 21–31. 10.1007/3-540-47977-5_2

[B12] Donoghue O. A. Harrison A. J. Coffey N. Hayes K. (2008). Functional data analysis of running kinematics in chronic Achilles tendon injury. Med. Sci. sports Exerc. 40 (7), 1323–1335. 10.1249/mss.0b013e31816c4807 18580414

[B13] Dreischarf M. Rohlmann A. Zhu R. Schmidt H. Zander T. (2013). Is it possible to estimate the compressive force in the lumbar spine from intradiscal pressure measurements? A finite element evaluation. Med. Eng. Phys. 35 (9), 1385–1390. 10.1016/j.medengphy.2013.03.007 23570899

[B14] Gagnon D. Larivière C. Loisel P. (2001). Comparative ability of EMG, optimization, and hybrid modelling approaches to predict trunk muscle forces and lumbar spine loading during dynamic sagittal plane lifting. *Clin. Biomech.* (Bristol, Avon) 16 (5), 359–372. 10.1016/s0268-0033(01)00016-x 11390042

[B15] Genaidy A. M. Waly S. M. Khalil T. M. Hidalgo J. (1993). Spinal compression tolerance limits for the design of manual material handling operations in the workplace. Ergonomics 36 (4), 415–434. 10.1080/00140139308967899 8472689

[B16] Godwin A. Takahara G. Agnew M. Stevenson J. (2010). Functional data analysis as a means of evaluating kinematic and kinetic waveforms. Theor. Issues Ergon. Sci. 11 (6), 489–503. 10.1080/14639220903023368

[B17] Granata K. P. Marras W. S. (1993). An EMG-assisted model of loads on the lumbar spine during asymmetric trunk extensions. J. Biomech. 26 (12), 1429–1438. 10.1016/0021-9290(93)90093-t 8308047

[B18] Hasegawa Y. Muramatsu M. (2013). “Wearable lower-limb assistive device for physical load reduction of caregiver on transferring support,” in IEEE/ASME international conference on advanced intelligent mechatronics, 1027–1032. 10.1109/AIM.2013.6584229

[B19] Hof A. L. (1992). An explicit expression for the moment in multibody systems. J. Biomech. 25 (10), 1209–1211. 10.1016/0021-9290(92)90076-d 1400520

[B20] Huysamen K. de Looze M. Bosch T. Ortiz J. Toxiri S. O’Sullivan L. W. (2018). Assessment of an active industrial exoskeleton to aid dynamic lifting and lowering manual handling tasks. Appl. Ergon. 68, 125–131. 10.1016/j.apergo.2017.11.004 29409626

[B21] ISO (2014). Robots and robotic devices – safety requirements for personal care robots.

[B22] ISO 11228-1:2021 (2021). Ergonomics — manual handling — Part 1: lifting, lowering and carrying.

[B23] Khalaf K. A. Parnianpour M. Sparto P. J. Barin K. (1999). Feature extraction and quantification of the variability of dynamic performance profiles due to the different sagittal lift characteristics. IEEE Trans. Rehabil. Eng. 7 (3), 278–288. 10.1109/86.788465 10498374

[B24] Kim H. K. Zhang Y. (2017). Estimation of lumbar spinal loading and trunk muscle forces during asymmetric lifting tasks: application of whole-body musculoskeletal modelling in OpenSim. Ergonomics 60 (4), 563–576. 10.1080/00140139.2016.1191679 27194401

[B25] Kobayashi H. Aida T. Hashimoto T. (2009). Muscle suit development and factory application. Int. J. Autom. Technol. 3 (6), 709–715. 10.20965/ijat.2009.p0709

[B26] Koo T. K. Li M. Y. (2016). A guideline of selecting and reporting intraclass correlation coefficients for reliability research. J. Chiropr. Med. 15 (2), 155–163. 10.1016/j.jcm.2016.02.012 27330520 PMC4913118

[B27] Koopman A. S. Kingma I. Faber G. S. De Looze M. P. van Dieën J. H. (2019a). Effects of a passive exoskeleton on the mechanical loading of the low back in static holding tasks. J. Biomech. 83, 97–103. 10.1016/j.jbiomech.2018.11.033 30514627

[B28] Koopman A. S. Toxiri S. Power V. Kingma I. van Dieën J. H. Ortiz J. (2019b). The effect of control strategies for an active back-support exoskeleton on spine loading and kinematics during lifting. J. Biomech. 91, 14–22. 10.1016/j.jbiomech.2019.04.044 31122661

[B29] Kozinc Ž. Baltrusch S. Houdijk H. Šarabon N. (2020). Reliability of a battery of tests for functional evaluation of trunk exoskeletons. Appl. Ergon. 86, 103117. 10.1016/j.apergo.2020.103117 32342882

[B30] Kudo N. Yamada Y. Ito D. (2019). Age-related injury risk curves for the lumbar spine for use in low-back-pain prevention in manual handling tasks. ROBOMECH J. 6, 12. 10.1186/s40648-019-0139-9

[B31] Lamers E. P. Yang A. J. Zelik K. E. (2018). Feasibility of a biomechanically-assistive garment to reduce low back loading during leaning and lifting. IEEE Trans. Biomed. Eng. 65 (8), 1674–1680. 10.1109/TBME.2017.2761455 28991732 PMC8820216

[B32] Luger T. Bär M. Seibt R. Rimmele P. Rieger M. A. Steinhilber B. (2021). A passive back exoskeleton supporting symmetric and asymmetric lifting in stoop and squat posture reduces trunk and hip extensor muscle activity and adjusts body posture–A laboratory study. Appl. Ergon. 97, 103530. 10.1016/j.apergo.2021.103530 34280658

[B33] Marras W. S. Jorgensen M. J. Davis K. G. (2000). Effect of foot movement and an elastic lumbar back support on spinal loading during free-dynamic symmetric and asymmetric lifting exertions. Ergonomics 43 (5), 653–668. 10.1080/001401300184314 10877482

[B34] Marras W. S. Lavender S. A. Leurgans S. E. Rajulu S. L. Allread W. G. Fathallah F. A. (1993). The role of dynamic three-dimensional trunk motion in occupationally-related low back disorders. The effects of workplace factors, trunk position, and trunk motion characteristics on risk of injury. *Spine* (Phila Pa 1976) 18 (5), 617–628. 10.1097/00007632-199304000-00015 8484154

[B35] Merryweather A. S. Loertscher M. C. Bloswick D. S. (2009). A revised back compressive force estimation model for ergonomic evaluation of lifting tasks. Work 34 (3), 263–272. 10.3233/WOR-2009-0924 20037241

[B36] Moudy S. Richter C. Strike S. (2018). Landmark registering waveform data improves the ability to predict performance measures. J. biomechanics 78, 109–117. 10.1016/j.jbiomech.2018.07.027 30126719

[B37] Nabeshima C. Ayusawa K. Hochberg C. Yoshida E. (2018). Standard performance test of wearable robots for lumbar support. IEEE Robot. Autom. Lett. 3 (3), 2182–2189. 10.1109/LRA.2018.2810860

[B38] Norman R. Wells R. Neumann P. Frank J. Shannon H. Kerr M. (1998). A comparison of peak vs cumulative physical work exposure risk factors for the reporting of low back pain in the automotive industry. Clin. Biomech. 13 (8), 561–573. 10.1016/S0268-0033(98)00020-5 11415835

[B39] Omoniyi A. Trask C. Milosavljevic S. Thamsuwan O. (2020). Farmers’ perceptions of exoskeleton use on farms: finding the right tool for the work(er). Int. J. Ind. Ergon. 80, 103036. 10.1016/j.ergon.2020.103036

[B40] Poliero T. Lazzaroni M. Toxiri S. Di Natali C. Caldwell D. G. Ortiz J. (2020). Applicability of an active back-support exoskeleton to carrying activities. Front. Robot. Ai. 7, 579963. 10.3389/frobt.2020.579963 33501340 PMC7805869

[B41] Potvin J. R. (1997). Use of NIOSH equation inputs to calculate lumbosacral compression forces. Ergonomics 40 (7), 691–707. 10.1080/001401397187847

[B42] Ramsay J. O. Munhall K. G. Gracco V. L. Ostry D. J. (1996). Functional data analyses of lip motion. J. Acoust. Soc. Am. 99, 3718–3727. 10.1121/1.414986 8655803

[B43] Ramsay J. O. Silverman B. W. (2005). “Fitting differential equations to functional data: principal differential analysis,” in Functional data analysis (New York: Springer), 327–348. 10.1007/0-387-22751-2_19

[B44] Sadler E. M. Graham R. B. Stevenson J. M. (2011). The personal lift-assist device and lifting technique: a principal component analysis. Ergonomics 54 (4), 392–402. 10.1080/00140139.2011.556259 21491281

[B45] Schultz A. Andersson G. Ortengren R. Haderspeck K. Nachemson A. (1982). Loads on the lumbar spine. Validation of a biomechanical analysis by measurements of intradiscal pressures and myoelectric signals. J. Bone Jt. Surg. Am. 64 (5), 713–720. 10.2106/00004623-198264050-00008 7085696

[B46] Stellato B. Banjac G. Goulart P. Bemporad A. Boyd S. (2020). OSQP: an operator splitting solver for quadratic programs. Math. Prog. Comp. 12 (4), 637–672. 10.1007/s12532-020-00179-2

[B47] Sugimoto Y. Naniwa K. Osuka K. (2011). “Static and dynamic characteristics of McKibben pneumatic actuator for realization of stable robot motions,” in Conference on intelligent robots and systems. Editor International R. S. J. (IEEE Publications), 1817–1822. 10.1109/IROS.2011.6094792

[B48] Tanaka M. Umeno S. Kikuchi Y. (2020). Development of a performance-testing method for a power assist suit designed for agricultural work. J. Jpn. Soc. Agric. Mach. 82, 196–198. 10.11357/jsamfe.82.2_196

[B49] Tondu B. (2012). Modelling of the McKibben artificial muscle: a review. J. Intell. Mater. Syst. Struct. 23, 225–253. 10.1177/1045389X11435435

[B50] Ulrey B. L. Fathallah F. A. (2013). Effect of a personal weight transfer device on muscle activities and joint flexions in the stooped posture. J. Electromyogr. Kinesiol. 23 (3), 195–205. 10.1016/j.jelekin.2012.08.014 23021604

[B51] Upasani S. Franco R. Niewolny K. Srinivasan D. (2019). The potential for exoskeletons to improve health and safety in agriculture—perspectives from service providers. IISE Trans. Occup. Ergon. Hum. Factors. 7 (3–4), 222–229. 10.1080/24725838.2019.1575930

[B52] van Dieën J. H. Faber G. S. Loos R. C. Kuijer P. P. F. Kingma I. van der Molen H. F. (2010). Validity of estimates of spinal compression forces obtained from worksite measurements. Ergonomics 53 (6), 792–800. 10.1080/00140131003675091 20496245

[B53] Waters T. R. Putz-Anderson V. Garg A. Fine L. J. (1993). Revised NIOSH equation for the design and evaluation of manual lifting tasks. Ergonomics 36 (7), 749–776. 10.1080/00140139308967940 8339717

[B54] Weston E. B. Alizadeh M. Knapik G. G. Wang X. Marras W. S. (2018). Biomechanical evaluation of exoskeleton use on loading of the lumbar spine. Appl. Ergon. 68, 101–108. 10.1016/j.apergo.2017.11.006 29409622

[B55] Whitfield B. H. Costigan P. A. Stevenson J. M. Smallman C. L. (2014). Effect of an on-body ergonomic aid on oxygen consumption during a repetitive lifting task. Int. J. Ind. Ergon. 44 (1), 39–44. 10.1016/j.ergon.2013.10.002

[B56] Wilke H. J. Neef P. Caimi M. Hoogland T. Claes L. E. (1999). New *in vivo* measurements of pressures in the intervertebral disc in daily life. Spine 24 (8), 755–762. 10.1097/00007632-199904150-00005 10222525

[B57] Wilke H. J. Neef P. Hinz B. Seidel H. Claes L. (2001). Intradiscal pressure together with anthropometric data–a data set for the validation of models. Clin. Biomech. 16, 111–126. 10.1016/S0268-0033(00)00103-0 11275349

[B58] Winter D. A. (2009). Biomechanics and motor control of human movement. New Jersey: John Wiley & Sons.

[B59] Xu Y. Qiu Y. Schnable J. C. (2018). Functional modeling of plant growth dynamics. Plant Phenome J. 1 (1), 1–10. 10.2135/tppj2017.09.0007

[B60] Zheng L. Hawke A. L. Evans K. (2022). Critical review on applications and roles of exoskeletons in patient handling. Int. J. industrial ergonomics 89, 103290. 10.1016/j.ergon.2022.103290 PMC9345507 35924209

